# Interplay between bladder microbiota and overactive bladder symptom severity: a cross‐sectional study

**DOI:** 10.1186/s12894-022-00990-0

**Published:** 2022-03-19

**Authors:** Kun Li, Chunxiao Chen, Jiarong Zeng, Yuehui Wen, Weihong Chen, Jie Zhao, Peng Wu

**Affiliations:** 1 Department of Urology, Nanfang Hospital, Southern Medical University, Guangzhou, China; 2 Department of Urology, Meizhou People’s Hospital, Meizhou, China; 3 School of Pharmaceutical Sciences, Southern Medical University, Guangzhou, China; 4 Clinical Microbiota Center, Nanfang Hospital, Southern Medical University, Guangzhou, China

**Keywords:** Overactive bladder, Urinary microbiome, Bladder microbiota, Severity of OAB symptom

## Abstract

**Background:**

It is widely accepted that there exist microbiota communities in urinary tract of healthy individuals. Imbalance in the urinary microbiome plays important roles in the development of various benign urological conditions including lower urinary track symptoms (LUTS) and overactive bladder (OAB). However, whether alteration in urinary microbiome exerts influence on the severity of OAB symptom has yet to be elucidated. The purpose of this study is to examine the correlation between urinary microbiome and the severity of OAB.

**Methods:**

A total of 70 OAB patients were recruited to finish overactive bladder symptom score (OABSS) questionnaires. Catheterized urine samples were obtained for 16S rRNA gene sequencing. The species richness and evenness were evaluated by α diversity, and the difference in urinary microbiome between patients with mild or moderate/severe severity was evaluated by β diversity. The relationship between urinary microbiome and the severity of OAB symptom was evaluated using Pearson’s correlation analysis.

**Results:**

Mild patients (OABSS ≤ 5, n = 17) had lower bacterial diversity (Simpson index, *P* = 0.024) and richness (Chao1, *P* = 0.023) than those with moderate/severe symptom (OABSS > 5, n = 53). Beta-diversity of urinary microbiome between two groups were significantly different. Furthermore, the score of OABSS was positively correlated with the richness index (Chao1,* P* = 0.002) and diversity index (Shannon index, *P* = 0.044) of urinary microbiome. Certain bacterial genera (e.g., *Porphyromona* and *Prevotella*) were significantly correlated with severity of OAB sub-symptoms.

**Conclusion:**

This study demonstrated that urinary microbiome was intimately correlated with the severity of OAB symptom and the increase of the diversity and richness of urinary microbiome was accompanied by more severe OAB symptoms, indicating that urinary dysbiosis may play pivotal roles in the deterioration of functional bladder diseases.

## Background

In the past decades, the well-recognized credendum that urine is normally sterile has been overthrown [1–3]. There are a growing body of evidence that an imbalance in the bladder’s normal microbiome may play a part in development of lower urinary tract symptoms (LUTS) [4–8]. Karstens and their co-workers found that women suffered from urge urinary incontinence (UUI) had an markedly different urinary microbiome composition compared with asymptomatic healthy individuals [2, 6]. In addition, our previous study showed that dysbiosis of urinary microbiome was responsible for the development of overactive bladder (OAB) symptom [9]. Although the severe symptoms adversely affect the quality of life of OAB patients, the factors affecting the severity are still unclear. Current guidelines show that research of urinary microbiome may help guide optimal management of OAB. However, to date, whether the female urinary microbiome influence the severity of OAB has yet to be elucidated [10].

Thus, in order to investigate whether the urinary microbiota (e.g., diversity, overall community structure, and/or specific taxonomic group) exert profound influence on the severity of OAB. We used *16S* ribosomal RNA (rRNA) gene sequencing to analyze the female urinary microbiome, and further decipher the relationships between diversity, bacteria community structure, specific taxonomic group and demographic and clinical characteristics of OAB patients.

## Methods

### Subject recruitment and urine collection

This cross-sectional study began following the institutional research ethics committee of Nanfang Hospital approval. Participants gave verbal and written research consent for research purposes. Between June 2020 and December 2020, adult patients from Nanfang Hospital aged 18 or above diagnosed with OAB were recruited into this study. According to the 2010 ICS definition of OAB [11], without infection or other pathological changes, patients must complain of urinary urgency, with or without urgency incontinence, usually accompanied by frequency and nocturia. We excluded women who had urinary tract infection (based on urine culture), administration of antibiotic in the past four weeks, immunodeficiency, neurological bladder, genitourinary cancer, pelvic radiation, urinary stones, degree of untreated pelvic organ prolapse (POP) greater than stage II of POP-Q, or pregnancy. Every one of the participants was required to complete the Overactive Bladder Symptom Score (OABSS) [12], and divided into mild and moderate/severe group by total score and the higher the score, the more severe the OAB symptom. Scores ≤ 5 were defined as mild symptom, and Scores > 5 were defined as moderate/severe symptom.

Urine was collected by a trained and licensed medical practitioner using sterile technology and sterile catheter. Kept all urine obtained from catheterization at 4 °C, and immediately transferred to laboratory within one hour, centrifuged at 16,000 g for 10 min, and the obtained precipitate was kept at − 80 °C for further processing.

### Isolation of DNA, PCR amplification and 16S rRNA gene sequencing

In order to minimize contamination, DNA extraction was carried out in the laminar flow engine hood using the cell culture scheme provided by the DNasy Blood and Tissue Kit (Qiagen, Germany). The concentration of extracted DNA was measured by using a Nanodrop ND-1000 spectrophotometer (Thermo Electron Corporation, USA). The *16S* rRNA sequence was amplified by PCR with primer set for V3–V4 region. Extracted negative controls (no urine) and PCR negative controls (no templates) were used to evaluated the contribution of foreign DNA in the reagents. The Qiaquick PCR purification kit (Qiagen of Valencia, USA) was used to purify the final PCR product from the nucleotides and primers that had not been incorporated. The purified samples were normalized to the same concentration of DNA, and were sequenced by Illumina MiSeq sequencer (Illumina, USA).

### Statistical analysis

Continuous variables were described as means and standard deviations (SD) or medians and interquartile ranges (IQR). The classification variables were described as frequencies and percentages. Differences in baseline characteristics between cohorts were evaluated by t-tests and Mann–Whitney U test of continuous variables, and Pearson Chi-square and Fisher’s exact tests of categorical variables. Through bivariate correlation analyses, the direction and intensity of the relationship between the scores of each item in OABSS (e.g., Daytime frequency, Nighttime frequency, Urgency, Urgency incontinence) and bacterial abundance are detected. Statistical analysis was carried out by using the Statistical Package for Social Science (SPSS, version 21, USA). Statistical tests were based on the probability of two tails. And when P value is less than 0.05, the result is considered significant..

In order to create an operational taxonomic units (OTUs) table, the raw reads were first processed by wrapper package Quantitative Insights Into Microbial Ecology (QIIME). Through the open reference selection strategy with Uclust, the sequences were clustered into individual OTU at the default similarity of 97%, and then chimera detection was performed by using the program UCHIME. Ribosomal Database Project Classifier was used to align a single representative sequence from each clustered OTU to the SILVA database and the Greengenes database.

Alpha diversity, including the Observed species, Chao1, Shannon, Simpson, Abundance-Based Coverage Estimator (ACE) and Pielou’s index, was evaluated using QIIME. The Chao1, ACE and the Observed species were used to calculate richness, and the larger the value, the richer the samples. Pielou’s Index was applied to calculate the bacterial evenness, and Pielou’s Index ranks samples from 0 to 1, with 1 being completely even. Shannon and Simpson index combine interaction between richness and evenness, smaller Simpson index values indicate less diverse communities with lower richness and/or evenness, while Shannon diversity is the opposite. Wilcoxon rank-sum test was applied to evaluated the difference of alpha diversity. Beta-diversity, measured by calculating the Bray Curtis, weighted UniFrac and unweighted UniFrac distances, was used to compare microbial composition between samples. Taxa summaries were reformatted and inputted into Linear discriminant analysis effect size (LEfSe) via the Huttenhower Lab Galaxy Server to identify significantly different bacteria as biomarkers between groups at the genus level. In the settings of LEfSe [13], the significantly specific bacteria were identified using the Mann–Whitney U test, and their effect size was estimated via linear discriminant analysis (LDA). The threshold value of logarithmic LDA score for discriminative features was 2.0.

## Results

### Participant demographic characteristics and clinical symptoms

82 female patients with OAB symptoms were recruited, however, twelve patients (mild = 4, moderate = 7, severe = 1) were excluded because bacterial DNA concentration extracted from their urine was too low to detect. Finally, A total of 70 female patients were enrolled and divided into mild symptom group (M group, N = 17) and the moderate/severe symptom group (M/S group, N = 53) based on their OABSS scores. Therefore,moderate/severe patients had significantly higher OABSS scores than mild patients. Standard urine culture were performed for all these patients to eliminate urinary infection. The demographic characteristics between the two groups were matched, including BMI, history of pelvic surgery, pregnancy and estrogen treatment (Table 1). In detail, mean age of the M group and the M/S group was 38.4 years and 37.1 years, respectively (*P* = 0.675). No significant difference in marriage and menopausal status was observed between 2 groups (marriage:* P* = 0.498, menopausal status: *P* = 1.000).

**Table1:** Characteristics of the study population

Characteristic	M group (n = 17)	M/S group (n = 53)	*P*-value
Age (years)	37.1 (11.4)	38.5 (11.6)	0.675^*a*^
BMI (kg/m^2^)	21.1 (2.3)	22.1 (3.1)	0.214^*a*^
Race/ethnicity			
Asian	17	53	NS
Other	0	0	
Currently married	15 (88.2)	42 (79.2)	0.498^*b*^
Ever pregnant	12 (70.6)	41 (77.4)	0.746^*b*^
Menopausal status			1.000^*b*^
Premenopausal	12 (70.6)	39 (73.6)	
Postmenopausal	5 (29.4)	14 (26.4)	
Use of hormone replacement therapy	0	6 (11.3)	0.175^*b*^
Diabetes	0	5 (9.4)	0.325^*b*^
Hypertension	2 (11.8)	10 (18.9)	0.717^*b*^
Pelvic surgery	2 (11.8)	16 (30.2)	0.203^*b*^
OABSS	4.47 (0.8)	8.58 (2.1)	< 0.001

Values given as mean (standard deviation) or median (interquartile range) and n (%)

Chi-square test used unless otherwise indicated

BMI, body mass index; OAB, overactive bladder; OABSS, overactive bladder Symptom Score

^a^Two sample t test

^b^Fisher’s exact test

### Sequencing data and relative abundances of urinary bacteria

**Fig. 1:**
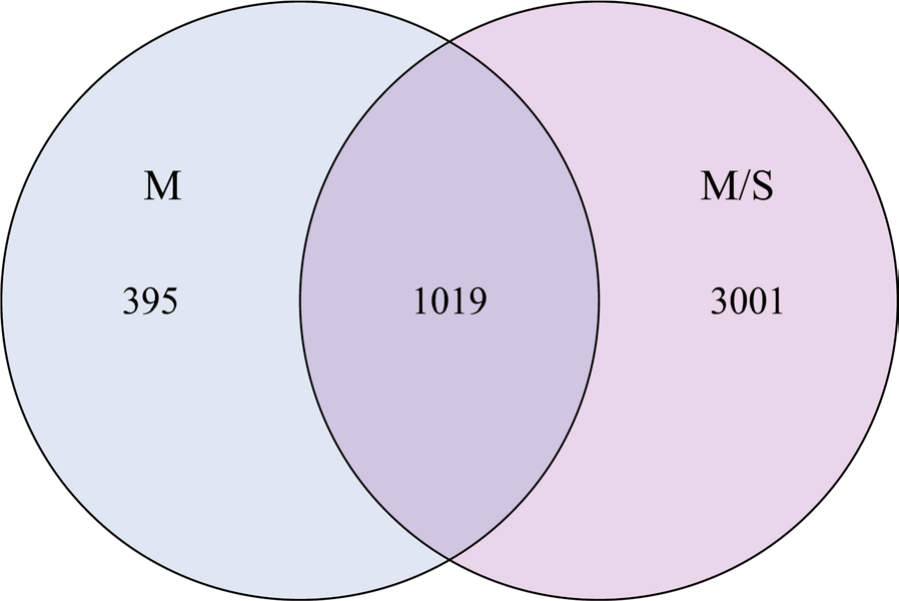
Venn diagram depicting the number of OTUs that are shared and unique between M group and M/S group

**Fig. 2:**
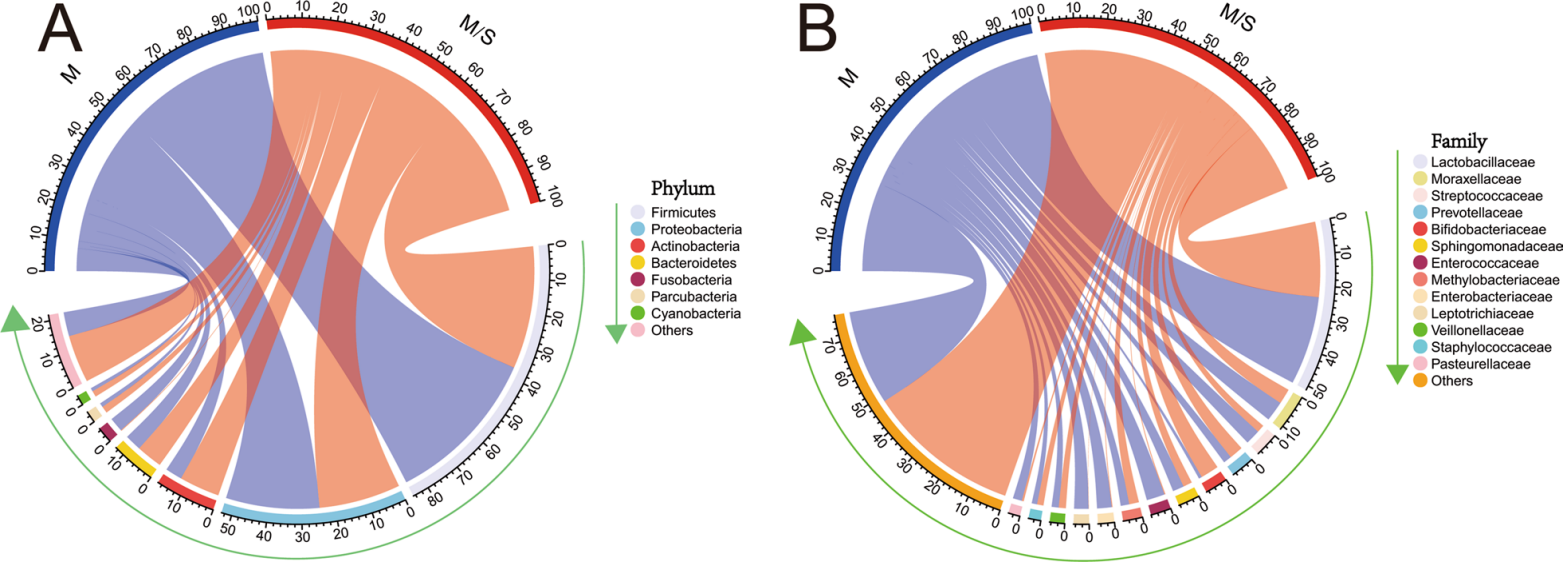
Bacterial relative abundance in M group and M/S group. Average distributions of 8 major phyla (**A**), 14 major families (**B**) are represented by circlize graphs. Each color represents a bacterial taxon which is displayed sequentially in the direction indicated by the arrow and the width of a colored ribbon represents the relative abundance of the organism within the sample

We totally obtained 3,926,088 sequences, 5,802,274 reads from the 70 samples. The median reads of the mild group and moderate/severe group was 77,177 and 78,329, respectively (*P* = 0.951). These reads were classified into 4415 OTUs for further analyses. Venn diagrams demonstrated that 1019 OTUs were common between two groups (Fig. 1). The median number of OTUs in the M/S group increased significantly (M group 177, M/S group 208, *P* = 0.043). These OTUs belong to 47 different Phyla and most OTUs were classified at the genus level. In addition, the relative abundance of taxa was used to analyze the composition of urinary microbiota at phylum and family levels (Fig. 2, Table 2). The most common phylum in both groups was Firmicutes (M group 47.7%, M/S group 37.0%), followed by Proteobacteria (M group 28.2%, M/S group 24.7%), Actinobacteria (M group 5.1%, M/S group 12.2%) and Bacteroidetes (M group 5.0%, M/S group 7.8%). Futhermore, phylum Actinobacteria was significantly more abundant in the moderate/severe group versus mild group (*P* = 0.018). Taxa from aforementioned four phyla were found in all patients at several levels. In detail, family of Lactobacillaceae, Veillonellaceae, Staphylococcaceae, Enterococcaceae and Streptococcaceae (Firmicutes phylum), Moraxellaceae, Methylobacteriaceae and Sphingomonadaceae (Proteobacteria phylum), Bifidobacteriaceae (Actinobacteria phylum), Prevotellaceae (Bacteroidetes phylum) were abundant in both groups with mean abundances > 2%. As shown in Table 2, only the family Bifidobacteriaceae was significantly more abundant in the mild groupcompared to the moderate/severe group. (*P* = 0.04). At the genus level, the mean abundances of 7 genera was > 2% in both groups, including Lactobacillus and Streptococcus (Firmicutes phylum), Gardnerella (Actinobacteria phylum), Prevotella (Bacteroidetes phylum), Methylobacterium, Acinetobacter and Sphingomonas (Proteobacteria phylum).

**Table 2:** Comparisons of mean relative abundances of bacteria (as percent reported by phyla: family)

Relative abundance (%)	M group (n = 17)	M/S group (n = 53)	*P*-value
Firmicutes	47.7	37.0	0.204
Lactobacillaceae	27.8	23.5	0.620
Veillonellaceae	2.2	2.2	0.992
Staphylococcaceae	1.7	2.0	0.732
Enterococcaceae	5.7	0.3	0.600
Streptococcaceae	4.6	2.4	0.641
Proteobacteria	28.2	24.7	0.575
Moraxellaceae	6.3	4.7	0.538
Methylobacteriaceae	2.7	3.0	0.868
Sphingomonadaceae	3.6	2.8	0.847
Actinobacteria	5.1	12.2	0.019
Bifidobacteriaceae	1.0	5.7	0.040
Bacteroidetes	5.0	7.8	0.248
Prevotellaceae	2.6	4.3	0.462

### Comparison of urotype between patients with mild and moderate/severe symptom

**Fig. 3:**
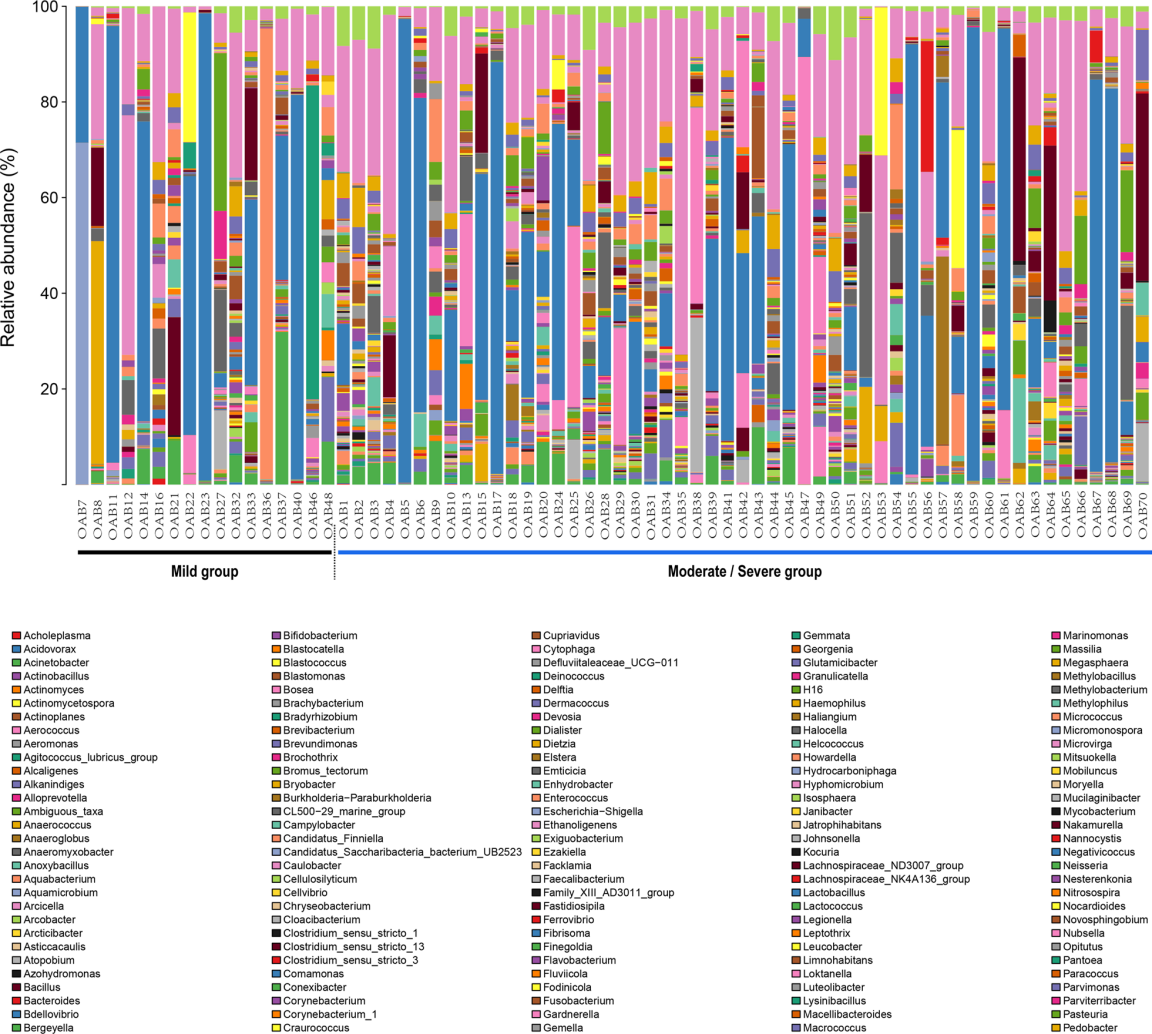
Overview of urinary microbiota of participants. Each bar corresponds to a subject and each colored box represents a genus. The height of each colored box indicates the relative abundance of corresponding bacteria in the sample

Urotype was defined by the predominant genus (relative abundance > 50%) present in urine from all OAB patients [2]. On the contrary, if none of the genus reaches a relative abundance of 50%, the urotype was regarded as “diverse”. The relative bacterial abundance of each OAB patients at the genus level was showed in Fig. 3. A total of 27 patients had specific urotypes, while urotypes of other patients were diverse. There were two urotypes in both groups (Lactobacillus and Streptococcus). The most common urotype in both groups was Lactobacillus (M group 29.4%, M/S group 22.6%). The urotypes of Haemophilus (OAB 8), Escherichia-Shigella (OAB 7), Enterococcus (OAB 36) and Sneathia (OAB 46) only appeared in the mild group, but there was no significant differences between the two groups (all *P* = 0.243). Similarly, although the Gardnerella urotype (OAB 47) was present only in the moderate/severe cohort, the difference was not statistically significant (*P* = 1.000). The number of unique bacterial genera in the urinary microbiome of each individual ranged from 24 to 117 (mild range 24–86 genera per sample, moderate/severe range 30–117 genera per sample).

### Alpha diversity

**Fig. 4:**
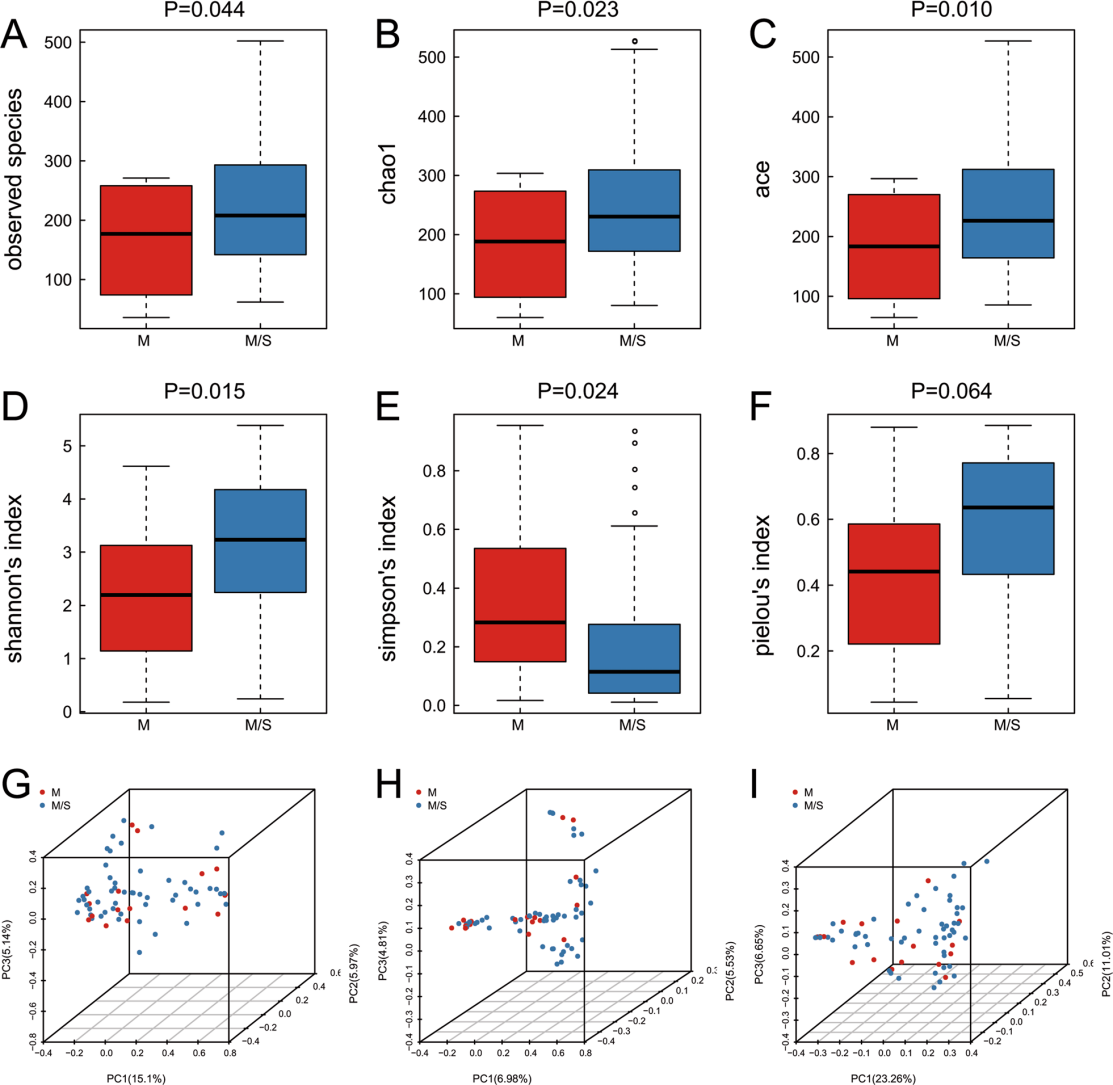
Alpha diversity and principal coordinate analysis for urinary microbiomes. Observed species (**A**); Chao1 (**B**); Ace (**C**); Shannon index (**D**); Simpson index (**E**); Pielou index (**F**). Principal coordinate analysis plot of the urinary microbiome based on the Bray–Curtis (**G**) and unweighted (**H**) or weighted (**I**) UniFrac distance metrics

Alpha diversity was measured by the Observed species, Chao1, Shannon, Simpson, ACE and Pielou index, which were described in the methods. The indices of bacterial alpha diversity were listed in Table 3. The Observed species, Chao1 and ACE (reflecting bacterial communities richness) were much higher in the moderate/severe cohort (Fig. 4A, Observed species, *P* = 0.044; Fig. 4B, Chao1, *P* = 0.023; Fig. 4C, ACE, *P* = 0.010). The Shannon index (Fig. 4D, *P* = 0.015) and Simpson index (Fig. 4E, *P* = 0.024) (reflecting bacterial overall diversity) were markedly lower in patients with mild symptoms. However, the Pielou index (Fig. 4F, *P* = 0.064) did not indicate a significant difference in bacterial evenness between the two groups.

**Table 3:** Comparisons of alpha diversity of microbiome

Parameter	M group (n = 17)	M/S group (n = 53)	*P*-value
Observed species	154.3 (90.7)	227.6 (122.4)	0.044
Chao1	178.1 (92.0)	257.1 (120.1)	0.023
Ace	181.9 (85.2)	265.8 (117.2)	0.010
Shannon index	2.1 (1.3)	3.1 (1.3)	0.015
Simpson index	0.4 (0.3)	0.2 (0.2)	0.024
Pielou index	0.4 (0.3)	0.6 (0.2)	0.064

Values given as mean (standard deviation)

OAB, overactive bladder

**Fig. 5:**
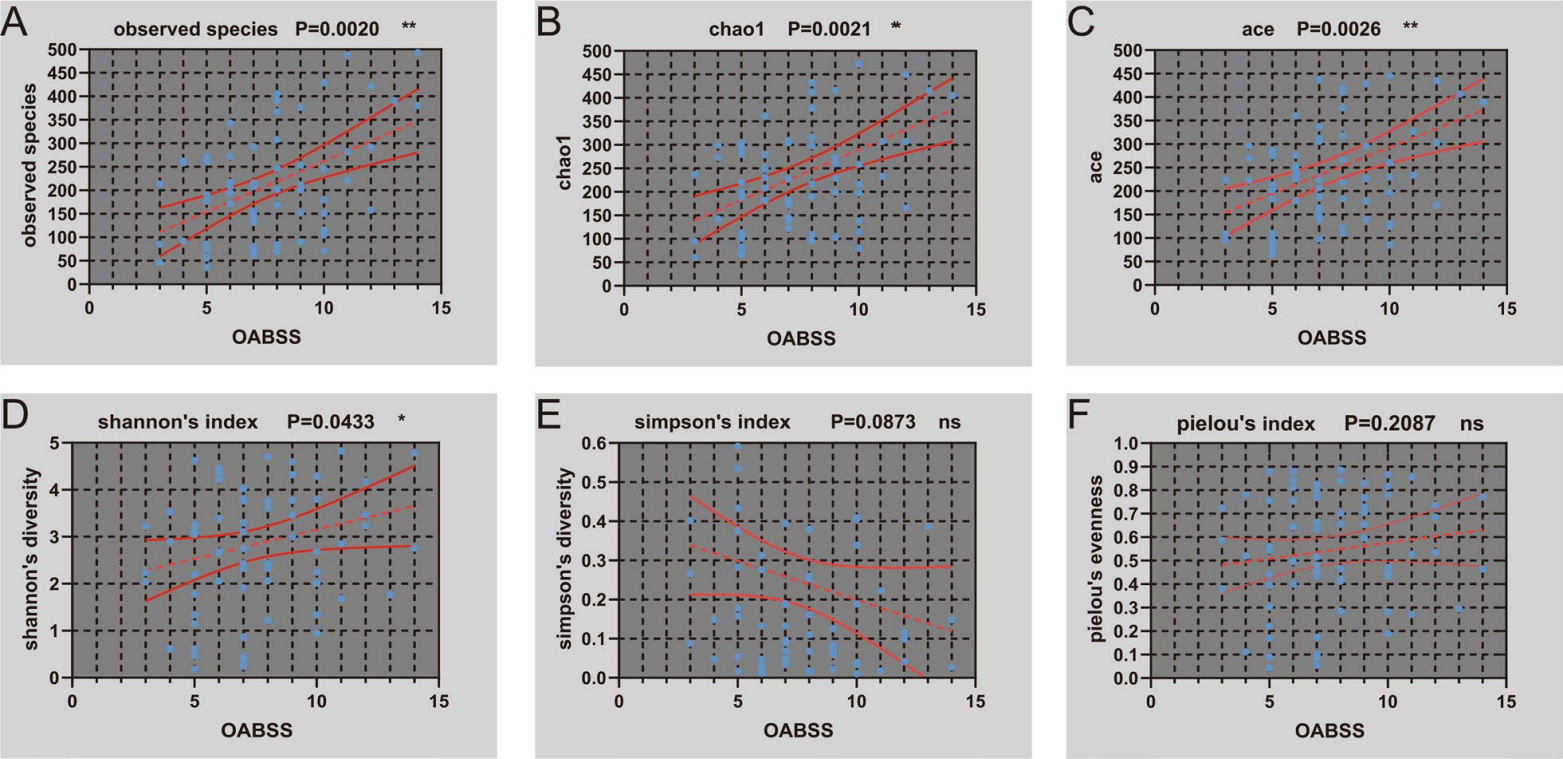
Correlation between alpha diversity and OABSS. Observed species (**A**); Chao1 (**B**); Ace (**C**); Shannon index (**D**); Simpson index (**E**); Pielou index (**F**)

In addition, Observed species, Chao1, ACE, and Shannon's Indices were significantly correlated to OABSS scores of the all OAB patients (Fig. 5A–D), and Simpson's Index (Fig. 5E) did not approach significance of this trend. And there was no correlation between Pielou's index and OABSS scores (Fig. 5F). Therefore, we speculated severity of OAB symptoms were correlated with richness, but not evenness.

### Beta diversity

Principal coordinate analysis (PCoA) was performed to compare urinary microbiota composition between the two cohorts based on unweighted UniFrac, weighted UniFrac, and Bray–Curtis distance metrics. (Fig. 4G–I). ANOSIM test revealed that there was significant difference in urinary microbiota composition between mild and moderate/severe groups using the Bray–Curtis distance metric (*P* = 0.021). However, no significant difference in bacterial composition was observed between two groups based on unweighted UniFrac distance metric (*P* = 0.22) and weighted UniFrac distance metric (*P* = 0.258).

### Specific genera associated with severity or sub-symptoms of OAB

Specific bacterial genera associated with OAB severity were identified using the LEfSe algorithm, however, no specific different genera were found between the two groups. The Pearson’s correlation analysis was performed to identify the specific bacterial genera related to sub-symptoms of OAB. Intriguingly, eight genera showed significant correlation with scores of the OABSS sub-symptoms. In detail, as shown in Table 4, one taxa were correlated with Daytime frequency, four taxa were related to Nighttime frequency, two taxa were correlated with Urgency, and two taxa in relation to Urgency incontinence. The strongest correlation was found between Bosea and Nighttime frequency (Correlation coefficient = 0.472, *P* < 0.001).

**Table 4:** Some genera of bladder microbiome were associated with specific OAB symptoms

Genus	OAB symptom	Correlation coefficient	*P*-value
Flavobacterium	Daytime frequency	0.342	0.004
Campylobacter	Nighttime frequency	0.258	0.031
Porphyromonas	Nighttime frequency	0.299	0.012
Prevotella	Nighttime frequency	0.291	0.015
Bosea	Nighttime frequency	0.472	< 0.001
Ezakiella	Urgency	0.280	0.019
Porphyromonas	Urgency	0.239	0.046
Bacillus	Urgency incontinence	0.298	0.012
Massilia	Urgency incontinence	0.313	0.008

OAB, overactive bladder

## Discussion

Since microorganisms in the bladder of female without UTI were detected [3], accumulating evidence has clearly confirmed that a living bacterial community inhabited in the bladder of women with LUTS and healthy individuals [2, 9, 14–16] Therefore,it is speculated that the urinary flora of women may play pivotal roles in lower urinary tract health and disease. In this cross-sectional study, we characterized the differences of urinary microbiomes between the women with different severity of OAB symptoms using high-throughput sequencing of the bacterial 16S rRNA gene.

In our data, the diversity and richness of bacteria in the moderate/severe patients' urine increased. In contrast, the evenness, which represents the distribution of microbial species, was not statistically different between M and M/S patients. We further found statistical difference in species diversity and richness between two groups based on the OABSS scores, and confirmed that higher the scores of OABSS were accompanied with the higher indices, including Observed species, Chao1, ACE, and Shannon’s index. Our results confirmed that more severe the symptoms of OAB patients with higher the diversity and richness of urine bacteria. Our data were consistent with the finding of PRICE [17], while inconsistent with the findings of Karstens L [6]. PRICE et al. used an enhanced culture method called Expanded Quantitative Urine Culture (EQUC) coupled with MALDI-TOF mass spectrometry and revealed that Chao1, ACE, and Shannon’s Indices were positively correlated to Urinary Distress Inventory (UDI) subscale score, which reflects the severity of the disease [18]. By contrast, Karstens et al. found that decreased microbial diversity in women with UUI was associated with increased symptom severity [6]. We speculated this discrepancy may be attributed to two completely different microbiome structural changes in two distinct disease state. It is elusive whether there exists an optimal composition of urinary microbiome, microbiome with too low or too high bacterial diversity are unlikely to be hemostasis. Low bacterial diversity may be associated with a lack of beneficial microbes and high diversity may reflect the lack of the urinary flora regulation mechanism. Although the composition of urinary microbiota is different in different individuals, patients with the same severity have similar urinary microbiota. As shown in the PCoA analysis, mild and moderate/severe groups clustered separately (Fig. 4), indicating that common urinary microbiota were associated with degree of OAB severity.

At the phylum level, we detected a significant increase of Actinobacteria in the urine of moderate/severe patients, consistent with our previous research in which we found that OAB patients have significantly more Actinobacteria compared with asymptomatic patients [9]. These findings implicated that disordered microbial populations may play an important role in the pathogenesis of OAB. At the genus level, correlation analysis found that several specific bacteria are associated with different OAB sub-symptoms, some bacteria of which have also been reported to be associated with LUTS. The abundance of *Prevotella* was positively correlated with the degree of nocturia. Consistently, in Pearce et al.’s study [2], they found that enrichment of *Prevotella* in UUI patients. Emerging studies have shown that the increased abundance of *Prevotella* species was related to a variety of diseases, including periodontitis, bacterial vaginosis, rheumatoid arthritis, low-grade inflammation, etc. [19]. Apostolidis A et al. found bladder biopsies with signs of chronic inflammation in patients with OAB [20]. This suggests that the bladder microbiome may participate in the occurrence and development of LUTS by causing chronic inflammation. We also detected that the more severe symptoms of Nighttime frequency were accompanied with higher abundance of *Porphyromona*. The same alteration of *Porphyromona* abundance was observed in Shoskes et al.’s study, which revealed that chronic prostatitis/chronic pelvic pain syndrome (CP/CPPS) patients had enrichment of genus *Porphyromona* [21]. All these data raise the possibility that specific microbial patterns may be associated with a specific disease, providing a novel direction for future researches of urinary microbiology and the diagnosis and treatment strategy of LUTS.

The most common urotype in both groups was *Lactobacillus*, and similar results were observed by PRICE in UUI patients [2]. In addition, we found the moderate/severe group had a smaller proportion of patients with *Lactobacillus*-dominant urine, and previous studies found that the UUI cohort exhibited decreased *Lactobacillus* sequence abundances compared to control cohort [2], and *Lactobacillus* DNA was more common in asymptomatic women than OAB patients [18], what's more, women who developed a post-treatment UTI had fewer *Lactobacillus* sequences [7]. *Lactobacillus* is a well-known bacterial genus in female vagina. It can prevent vaginitis by maintaining the acidic physiological environment of the vagina. It is possible that *Lactobacillus* in the bladder also plays an important regulatory or protective role in functional lower urinary tract (LUT) disorders.

Our present study has several major advantages. First, we used catheterized urine specimens instead of voided midstream urine. Wolfe and his coworkers discovered that the microbiome between voided urine and urine collected by transurethral catheter were significantly different, and microbiome in catheterized urine specimens were more close to the bladder microbiome, without the contamination of microbial flora from the genital tract [3]. Second, except for the scores of OABSS, the demographic characteristics (e.g., age, BMI, race/ethnicity) of the two cohorts were matched. Therefore, many factors that cause the difference in urinary microbiota are excluded. Third, the total sample size of OAB patients included in this study is relatively large. In addition, all patients’ urine samples were subjected to standard urine culture to eliminate urinary tract infection. The study’s limitation was mild OAB patients were relatively few. Only OABSS was used in this study to assess the severity of the patients' symptoms, OABSS evaluates symptoms from the patients' viewpoint and does not require bladder diary recording. Therefore, the results may be greatly affected by the subjective feelings of patients. Furthermore, in this cross-sectional study, it is difficult to determine the causal relationship between symptoms and urinary microbiota. Thus, additional large-scale prospective studies are required to clarify the important role of the urinary microflora in the development of OAB.

## Conclusion

In conclusion, findings in our study suggest that the bladder microbiome is closely related to OAB severity. The results of our research confirmed that the higher bacterial diversity and richness was associated with worse OAB symptom severity. In addition, several specific bacterial genera related to sub-symptoms of OAB, suggesting that specific urinary dysbiosis may exacerbate functional bladder disorders. In addition, the urinary microbiota may have potential clinical diagnostic and therapeutic implications in the future.

## Data Availability

The 16S rRNA gene sequences are available in the NCBI SRA database, [https://www.ncbi.nlm.nih.gov/sra/PRJNA661243].

## Abbreviations

LDA: Linear discriminant analysis
LEfSe: Linear discriminant analysis effect size
LUTS: Lower urinary track symptoms
OAB: Overactive bladder
OABSS: Overactive bladder symptom score
OTUs: Operational taxonomic units
PCoA: Principal co-ordinate analysis
POP: Pelvic organ prolapse
QIIME: Quantitative insights into microbial ecology
